# Hypoxia and Exercise Increase the Transpulmonary Passage of ^99m^Tc-Labeled Albumin Particles in Humans

**DOI:** 10.1371/journal.pone.0101146

**Published:** 2014-07-11

**Authors:** Melissa L. Bates, Emily T. Farrell, Alyssa Drezdon, Joseph E. Jacobson, Scott B. Perlman, Marlowe W. Eldridge

**Affiliations:** 1 Department of Pediatrics, Critical Care Division and the John Rankin Laboratory of Pulmonary Medicine, University of Wisconsin School of Medicine and Public Health, Madison, Wisconsin, United States of America; 2 Department of Radiology, University of Wisconsin School of Medicine and Public Health, Madison, Wisconsin, United States of America; 3 Departments of Biomedical Engineering and Kinesiology, University of Wisconsin School of Medicine and Public Health, Madison, Wisconsin, United States of America; 4 Michigan State University College of Human Medicine, East Lansing, Michigan, United States of America; Indiana University, United States of America

## Abstract

Intrapulmonary arteriovenous anastomoses (IPAVs) are large diameter connections that allow blood to bypass the lung capillaries and may provide a route for right-to-left embolus transmission. These anastomoses are recruited by exercise and catecholamines and hypoxia. Yet, whether IPAVs are recruited via direct, oxygen sensitive regulatory mechanisms or indirect effects secondary to redistribution pulmonary blood flow is unknown. Here, we hypothesized that the addition of exercise to hypoxic gas breathing, which increases cardiac output, would augment IPAVs recruitment in healthy humans. To test this hypothesis, we measured the transpulmonary passage of ^99m^Tc-macroaggregated albumin particles (^99m^Tc-MAA) in seven healthy volunteers, at rest and with exercise at 85% of volitional max, with normoxic (FIO_2_ = 0.21) and hypoxic (FIO_2_ = 0.10) gas breathing. We found increased ^99m^Tc-MAA passage in both exercise conditions and resting hypoxia. However, contrary to our hypothesis, we found the greatest ^99m^Tc-MAA passage with resting hypoxia. As an additional, secondary endpoint, we also noted that the transpulmonary passage of ^99m^Tc-MAA was well-correlated with the alveolar-arterial oxygen difference (A-aDO_2_) during exercise. While increased cardiac output has been proposed as an important modulator of IPAVs recruitment, we provide evidence that the modulation of blood flow through these pathways is more complex and that increasing cardiac output does not necessarily increase IPAVs recruitment. As we discuss, our data suggest that the resistance downstream of IPAVs is an important determinant of their perfusion.

## Introduction

Inducible intrapulmonary arteriovenous anastamoses (IPAVs) are large diameter conduits that bypass the pulmonary capillary network. The existence of these pathways has been previously demonstrated with saline contrast echocardiography [1]–[3] and ^99m^Tc-labeled albumin macroaggregates (^99m^Tc-MAA) [4] in humans and solid latex microspheres in animals [5], [6] and ex-vivo lung preparations [5]–[10]. While not typically perfused at rest, IPAVs open in ∼90% of humans performing moderate to heavy intensity exercise [1], [4]. These pathways are also recruited by hypoxia (FIO_2_ = 0.10–0.08) [6], [11] and their recruitment during exercise can be prevented or reversed by hyperoxia (FIO_2_ = 1.0) [12], [13]. Whether these observations are due to direct oxygen sensitive regulatory mechanisms or indirect effects secondary to redistribution pulmonary blood flow is unknown.

The mechanism by which IPAVs are recruited by exercise and hypoxia is not known. Several investigators have suggested that the transpulmonary passage of particles or bubbles through IPAVs is the result of elevated cardiac output and may occur in order to moderate pulmonary vascular pressure and decrease right ventricular stress [14]–[16]. Laurie, et al. observed right-to-left transpulmonary saline bubble passage through IPAVs in a group of healthy adults infused with epinephrine. The intensity of right-to-left bubble passage was correlated with cardiac output and these authors concluded that IPAVs recruitment was the result of a catecholamine-induced increase in cardiac output [13]. Additionally, Bryan, et al. demonstrated increases in cardiac output, intrapulmonary shunt (Q_S_/Q_T_), and transpulmonary bubble passage in healthy humans with dobutamine infusion [17]. Similarly, they found increased right-to-left bubble passage and also concluded that IPAVs recruitment was related to cardiac output.

It is possible, however, that the recruitment of IPAVs occurs only coincidentally with the elevation in cardiac output. The strongest evidence against a causative role for cardiac output is the fact that hyperoxia nearly universally reverses catecholamine-induced or exercise-induced IPAVs recruitment [12], [13]. Furthermore IPAVs recruitment can occur at rest with alveolar hypoxia with a minimal increase in cardiac output and pulmonary vascular pressure [11]. Indeed, these data provide compelling evidence that IPAVs regulation is in part mediated by an oxygen sensitive mechanism and not primarily by mechanical factors such as increases pulmonary blood flow and driving pressure.

Given that IPAVs recruitment has never been quantified in the context of hypoxia or hypoxic exercise, it is also unknown if IPAVs recruitment is similar to that observed under other conditions. If IPAVs recruitment and cardiac output are indeed linked, then we would predict that their recruitment by hypoxia would be further enhanced by the addition of exercise. To test this hypothesis, we measured the transpulmonary passage of ^99m^Tc-MAA particles at rest and at 85% of maximal exercise with normoxic and hypoxic gas breathing, according to previously described methods [4]. As a secondary endpoint, arterial blood gases were measured in each condition. By assessing IPAVs recruitment under these conditions, we are able to further contribute to our understanding of how these pathways are regulated. Surprisingly, and contrary to our hypothesis, we found the transpulmonary passage of ^99m^Tc-MAA to be greatest with resting hypoxic gas breathing.

## Methods

### Ethics statement

Participants gave written, informed consent. The consent procedure and materials and the experimental protocol were approved by the Institutional Review Board at the University of Wisconsin School of Medicine and Public Health.

### Subject population and screening

We recruited twelve individuals (18–35 years) from the general population of the University of Wisconsin-Madison. Data are available from the authors upon request.

Because a right-to-left intracardiac shunt could allow ^99m^Tc-MAA to bypass the lung independent of IPAVs, we screened participants for septal shunts, including a patent foramen ovale (PFO). To observe these shunts, a saline contrast echocardiogram with a Valsalva maneuver was performed prior to participation. The Valsalva maneuver was standardized such that each participant donned nose clips and exhaled against a fixed resistance to generate +40 cmH_2_O mouth pressure for 10–15 seconds, coinciding with the injection of saline contrast [18]. This pressure was then released and the transeptal passage of bubbles was assessed. This was repeated without Valsalva in order to exclude individuals with pathological pulmonary arteriovenous malformations. A PFO was observed in five individuals (42%) and they were excluded from participation.

Because ^99m^Tc-MAA is contraindicated in pulmonary hypertension [19], the peak velocity of the tricuspid regurgitation jet [20], [21] was evaluated in the remaining participants and found to be normal. Forced spirometry and single breath carbon monoxide diffusion capacity were measured using commercially-available equipment (Optima, Medgraphics, St. Paul, MN) using standardized techniques [22], [23]. Percent predicted values were calculated using previously published regression equations [24]–[26]. Pulmonary function equipment was calibrated prior to each use with a 3L syringe and manufacturer-supplied carbon monoxide and neon control gases.

### Overall study design

Participants visited the laboratory on five occasions with at least one week between visits. This interval was selected to ensure complete clearance of any previously-injected ^99m^Tc-MAA (^99m^Tc half-life = 6.0 hours). During visits 1&2, ^99m^Tc-MAA was injected intravenously after ten minutes of FIO_2_ = 0.21 or 0.10 breathing. During visits 3&4, ^99m^Tc-MAA was injected while the participant breathed either FIO_2_ = 0.21 or 0.10 while exercising at 85% of their maximal exercise capacity. On the 5^th^ visit, ^99m^Tc-MAA was again injected after ten minutes of FIO_2_ = 0.21 breathing. Because the resting FIO_2_ = 0.21 injection is used as a baseline by which changes in ^99m^Tc-MAA passage in the other conditions are compared [4], the 5^th^ visit served as a control in order to evaluate the reproducibility of transpulmonary ^99m^Tc-MAA passage.

### Preparation of the ^99m^Tc-MAA injection

Briefly, ^99m^Tc-MAA (Pulmolite, Pharmalucence, Bedford, MA) was prepared by a nuclear pharmacist such that each injection contained ∼300,000 particles with 3–4 miC of activity. The fraction of unbound ^99m^Tc was determined for each injection using thin-layer chromatography and was 4.8±2.8%. The same lot of MAA was used throughout the study. Microscopic analysis (ImageJ, National Institutes of Health, Bethesda, MD) of 1×10^6^ particles, sampled from three different vials revealed a mean particle diameter of 29 µm. with 97% of particles greater than 10 µm on the shortest axis, but none was greater than 120 µm.

We investigated the feasibility of removing particles <20 µm by vacuum filtration prior to injection (Steriflip, Millipore, Billerica, MA). Vacuum filtration was highly effective in removing particles <20 µm, but resulted in the formation of several particles >500 µm. Because the injection of particles >150 µm is not recommended in humans, we did not filter ^99m^Tc-MAA prior to injection.

### Study design – Visits 1, 2, & 5

A 22-gauge catheter was placed in an antecubital vein for the injection of ^99m^Tc-MAA. Participants then laid supine, donned nose clips, and breathed FIO_2_ = 0.21 or 0.10 (balance N_2_) through a mouthpiece fitted to a 2-way non-rebreathing valve. After three minutes, arterial blood was drawn via radial arterial puncture. Participants were then seated upright and continued breathing for an additional seven minutes. At the end of the breathing trial, ^99m^Tc-MAA was injected into the intravenous catheter. Whole body images were then acquired to quantify the fraction of ^99m^Tc-MAA bypassing the pulmonary circulation.

During visits 1&2, participants also performed a graded exercise test, on a magnetically-braked cycle ergometer (Velotron, RacerMate, Seattle, WA) while breathing either FIO_2_ = 0.21 or 0.10. Participants wore noseclips and a 2-way non-rebreathing mouthpiece for the measurement of ventilation and metabolic function. Beginning at 60 W, the workload was increased 25–50 W every five minutes. The exercise test was ended when the participant could no longer maintain a pedaling cadence ≥55 rpm for three minutes. Each test lasted 25–30 minutes. Stages longer than those of a typical max test were used to mirror the exercise performed in visits 3&4, where additional time was required to accommodate the ^99m^Tc-MAA injection and to allow sufficient time for complete circulation of the ^99m^Tc-MAA particles. A single ^99m^Tc-MAA injection requires about two minutes, including agitation of the material, injection, and thorough flushing of the infusion line and syringe. Thus, a workload thought to be maintainable for the full duration of the 5-minute stage (85% of max wattage) was chosen. An exercise test was not performed at visit 5. The purpose of visit 5 was to evaluate the reproducibility of the ^99m^Tc-MAA injection.

### Study design – Visits 3&4

Prior to exercise, participants swallowed a pill-shaped core temperature probe (CorTemp, HQ Inc., Palmetto, FL). A 22-gauge catheter was placed in an antecubital vein for ^99m^Tc-MAA injection and a 3F angiocath was placed in a brachial artery for blood sampling.

Participants were seated on the ergometer and fitted with noseclips. Ventilation and metabolic function were measured as in visits 1&2. After being seated at rest for 10 minutes, participants began exercising at 30% of the maximal wattage achieved during visit 1&2. They completed five minute stages at 30%, 45%, 60%, 75%, and 85% of maximal capacity. During the third minute of each stage, arterial blood gases were drawn. One participant during normoxic exercise and two participants during hypoxic exercise became exhausted prior to reaching 85% of max and measurements were made in the 75% stage. During the third minute of the final stage, ^99m^Tc-MAA was injected into the intravenous catheter such that the ^99m^Tc-MAA injection coincided with the drawing of arterial blood.

### Imaging Methods

We have previously discussed, in detail, the use of ^99m^Tc-MAA to detect IPAVs recruitment, including the limitations of this technique [4], and would direct the reader to this publication for further information. Participants were imaged supine with anterior and posterior whole body images acquired simultaneously by gamma camera (VPS-45, General Electric Medical Systems, Waukesha, WI) using a 20-minute “step and shoot” protocol. The anterior and posterior images were reconstructed into a single whole body image where each voxel represents the geometric mean of the anterior and posterior image (GE eNTEGRA software, GE Healthcare). The fraction of ^99m^Tc-MAA traversing the pulmonary circulation was calculated by quantifying one minus the lung-to-whole body ratio from this unified image and correcting it for the amount of unbound ^99m^Tc99. As described previously [4], the lung region of interest (ROI) was defined using the normoxic exercise scan and included all voxels with at least 3–5% of the peak voxel intensity. The ROI was then applied to the other images and visually inspected to ensure that the ROI contained the entirety of the lung. Because saline contrast and solid microsphere studies have repeatedly demonstrated that IPAVs are not recruited at rest in normoxia, the lung-to-whole body ratio in the normoxic resting condition was set as a baseline. Changes with hypoxia and exercise are described subsequently as relative to this baseline. Again, to verify the reproducibility of the resting normoxic injection, this procedure was repeated on a second occasion.

### Blood gas and metabolic measurements

For each sampling, arterial blood was drawn into three airtight, heparinized syringes. Each syringe was analyzed once (pHOx Basic, Nova Biomedical, Waltham, MA) immediately after collection and corrected for body temperature. No more than five minutes passed between the collection and analysis. A three-point calibration was done prior to the study using manufacturer-supplied control solutions.

Equipment used to measure metabolic and ventilatory parameters (Optima, Medgraphics, St. Paul, MN) was calibrated prior to use with a 3L syringe and manufacturer-supplied carbon dioxide and oxygen control gases.

Alveolar PO_2_ was calculated according to the following equation: PAO_2_ = PIO_2_–(PaCO_2_/R)+[FIO_2_×PaCO_2_×(1-R)/R], where PIO_2_ is the dry inspired partial pressure of oxygen and R is the respiratory exchange ratio [27]. The A-aDO_2_ was then calculated as the difference between the alveolar and arterial PO_2_.

### Statistical Analysis

All data are displayed as ± standard deviation unless otherwise noted. Blood gas and metabolic parameters were compared using repeated measures ANOVA with post hoc analysis (Student’s t-test) to compare differences between exercise and rest, as well as between normoxia and hypoxia. Paired t-tests were used to evaluate differences in ^99m^Tc-MAA passage with hypoxia and exercise. Significance was determined when p<0.05 with a Bonferroni correction applied for multiple comparisons. Predictors of the A-aDO_2_ during exercise were evaluated by repeated measures general linear model (Minitab, State College, PA) in which the participant was treated as a random variable, the FIO_2_ as a fixed variable, and the ^99m^Tc-MAA fraction as a covariate. Reproducibility of the baseline ^99m^Tc-MAA injection was assessed by calculating the intra-class correlation coefficient between the repeated visits and the repeatability coefficient [28]. If ^99m^Tc-MAA passage was beyond the repeatability coefficient, then it was concluded that the difference was outside of the measurement’s error range.

## Results

Anthropometric characteristics, pulmonary function, and exercise capacity data are given in Table 1.

**Table 1:** Anthropometric characteristics of the seven participants completing the study.

								VO_2_max	VO_2_max
	Age		Height	Weight	FEV_1_		DL_CO_	FIO_2_ = 0.21	FIO_2_ = 0.10
Subject	(yrs)	Sex	(cm)	(kg)	(L)	FEV_1_/FVC	(mL/min/Torr)	(mL/kg/min)	(mL/kg/min)
01	23	F	165	56	3.54	0.83	30.74	30.9	20.6
					(113%)		(97%)	(92.3)	
02	22	M	183	80	5.10	0.85	45.06	56.5	32.4
					(101%)		(119%)	(117.1)	
03	21	F	171	61	3.98	0.88	32.12	43.3	30.2
					(116%)		(95%)	(98.4)	
04	31	M	172	88	4.02	0.88	37.14	33.6	18.6
					(106%)		(109%)	(76.4)	
05	33	F	171	62	3.90	0.83	32.57	57.1	32.3
					(121%)		(96%)	(132.4)	
06	21	M	178	76	4.42	0.86	34.59	45.2	23.8
					(95%)		(96%)	(93.3)	
07	20	M	184	72	4.36	0.75	42.92	58.0	30.5
					(103%)		(101%)	(118.6)	
Mean ±	24±5		175±7	71±12	4.19±0.50	0.84±0.04	36.45±5.57	46.4±11.3	26.9±5.8*
SD					(108±9)		(102±9)	(104±19)	

FVC, forced vital capacity; FEV_1_, forced expired volume in 1 second; DL_CO_, diffusion capacity for carbon monoxide; VO_2_max; relative maximal oxygen uptake.

^*^indicates p<0.05. Values in parentheses are percent predicted (23–25).

### Baseline transpulmonary ^99m^Tc-MAA passage

**Figure 1:**
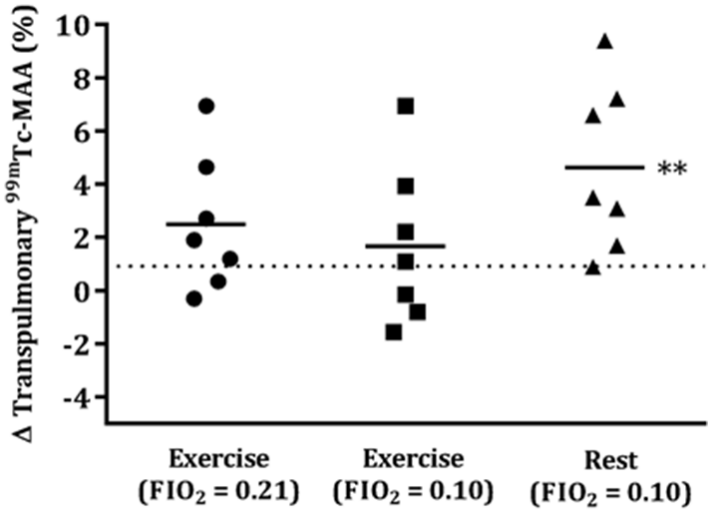
Change in the transpulmonary passage (%) of ^99m^Tc-MAA compared to resting, normoxic gas breathing. Dashed line indicates the repeatability coefficient (0.92%) Transpulmonary ^99m^Tc-MAA passage was noted in 6/7 participants performing exercise in normoxia and 4/7 participants performing exercise in hypoxia. Breathing hypoxic gas at rest increased ^99m^Tc-MAA passage in all participants relative to hypoxic exercise. ** indicates a difference compared to hypoxic rest (p = 0.001).

**Figure 2:**
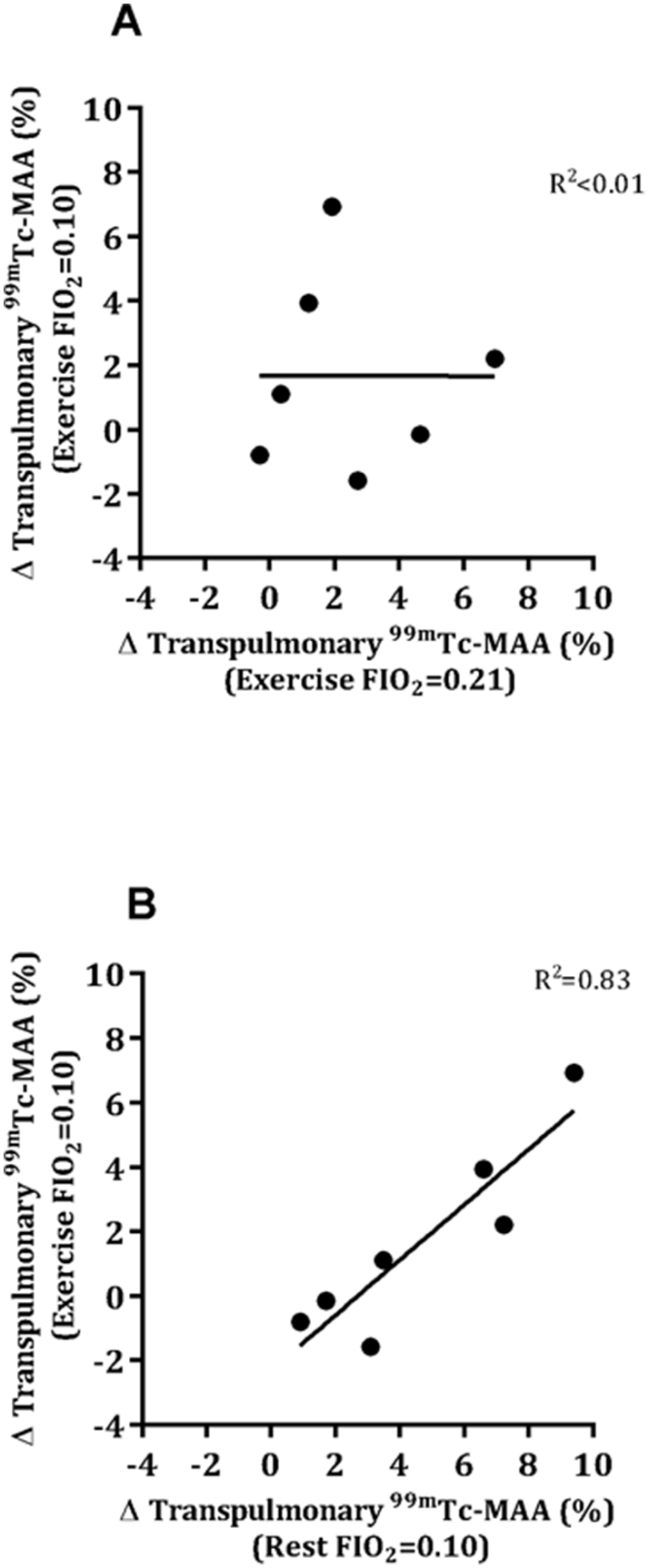
Relationship between transpulmonary ^99m^Tc-MAA passage with exercise in normoxia vs. hypoxia (A) and between rest and exercise in hypoxia (B). The transpulmonary passage of ^99m^Tc-MAA with exercise in hypoxia was well-correlated with that measured at rest with hypoxic gas breathing. Dashed line indicates the line of identity.

Transpulmonary passage of ^99m^Tc-MAA was 11.7±9.1% and 11.4±9.4% during repeated normoxic rest (visits 1 and 5, respectively) and were not statistically different (p = 0.41). This is consistent with the percentage of particles known to be ≤15 µm. The repeatability coefficient was 0.92%. As we have done previously [4], each individual’s resting value was used as a correction factor for all subsequent scans. At rest, hypoxia increased the passage of ^99m^Tc-MAA particles in 6/7 participants (Δ5.3±2.9%) (Figure 1). Normoxic exercise increased ^99m^Tc-MAA passage in 5/7 participants (71%) (Δ3.5±2.3%) and hypoxic exercise increased ^99m^Tc-MAA passage in 4/7 participants (57%) (Δ3.5±2.5). The overall population means in normoxic and hypoxic exercise were not different (Δ2.5±2.6% vs Δ1.7±3.0%, p = 0.60), nor were the percent changes in transpulmonary ^99m^Tc-MAA passage correlated between the two conditions (R^2^<0.01, p = 0.996), meaning that the same individuals did not necessarily recruit IPAVs similarly in both conditions (Figure 2A). While the percent changes in transpulmonary passage of ^99m^Tc-MAA for each individual were well-correlated between hypoxic rest and hypoxic exercise (R^2^ = 0.82, p = 0.005), the overall percentage of ^99m^Tc-MAA passage was higher with hypoxic rest than hypoxic exercise (Δ4.6±3.1% vs Δ2.5±2.6%, p = 0.001) (Figure 2B).

**Figure 3:**
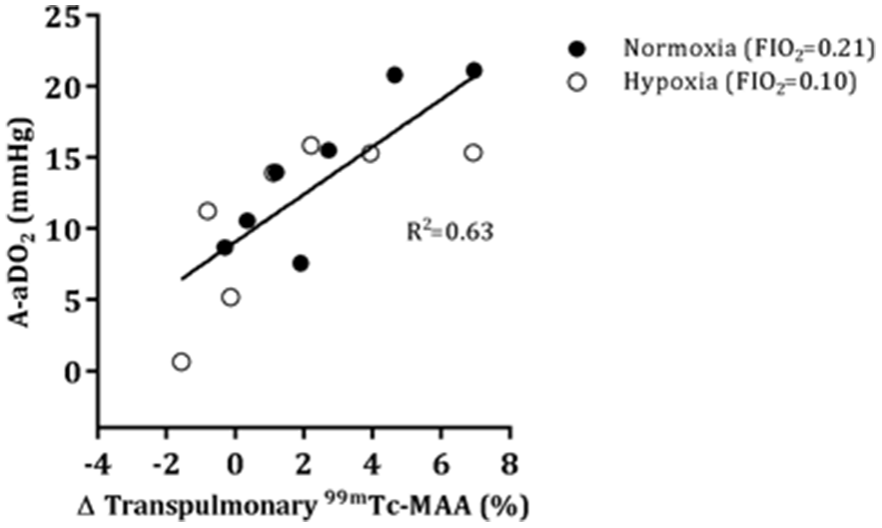
Relationship between transpulmonary ^99m^Tc-MAA passage and the alveolar-arterial oxygen difference (A-aDO_2_) in normoxic and hypoxic exercise. Line represents the result of a general linear model analysis in which the transpulmonary ^99m^Tc-MAA passage was linearly correlated with the A-aDO_2_ (R^2^ = 0.63), but this relationship was not dependent on the FIO_2_ (p>0.05).

Blood gas parameters for normoxic and hypoxic rest and exercise are given in Table 2. Exercise widened the A-aDO_2_, although there was no difference between normoxic and hypoxic exercise (p = 0.43). The A-aDO_2_ during the last exercise stage was significantly related to the fraction of transpulmonary ^99m^Tc-MAA passage (p = 0.01) and 63% of the variance in the A-aDO_2_ was accounted for by the percent passage of ^99m^Tc-MAA (Figure 3).

**Table 2:** Arterial blood gases, respiratory quotient (R), and the alveolar-arterial PO_2_ difference (A-aDO_2_), measured at rest and at 85% of the maximal attainable wattage during the resting and exercise visits.

	Condition	FIO_2_	R	pH	PCO_2_ [mmHg]	PO_2_ [mmHg]	A-aDO_2_ [mmHg]
Exercise Visit	Rest	0.21	0.86±0.11	7.46±0.02	38.8±4.9	102.1±8.3	−1.2±5.0
	Rest	0.10	1.34±0.15	7.48±0.02	34.3±3.9	43.8±3.6	−1.1±4.7
	85% Max Wattage	0.21	1.15±0.11*	7.38±0.07*	33.5±4.0*	101.3±5.7	14.1±5.2*
	85% Max Wattage	0.10	1.27±0.16*	7.39±0.07*	27.1±3.0* †	37.8±0.5* †	11.1±5.9*
Resting Visit	Rest	0.21	0.87±0.09	7.48±0.05	38.7±4.9	103.8±9.8	2.6±7.1
	Rest	0.10	1.19±0.11	7.48±0.01	37.9±3.3	42.1±5.0	−1.8±4.7

^*^indicates p<0.05 compared to values measured at rest on the same day.

† indicates p<0.05 compared to normoxic exercise.

## Discussion

Our interest in intrapulmonary arteriovenous anastomoses (IPAVs) stems from the finding that these large diameter pathways can be recruited in ∼90% of healthy individuals and may provide a conduit for right-to-left embolus transmission. Unlike our previous work investigating IPAVs recruitment with normoxic exercise [4], we have not directly quantified IPAVs recruitment with hypoxia or hypoxic exercise. To that end, our primary goal was to quantify the transpulmonary passage of ^99m^Tc-MAA particles in the face of hypoxia. We aimed to further understand factors that influence their recruitment by specifically asking if exercise universally increases IPAVs recruitment. Here, we provide evidence suggesting that exercise, which increases cardiac output, does not necessarily increase IPAVs recruitment. Indeed, if cardiac output were a major modulator of IPAVS recruitment in hypoxia, we would expect ^99m^Tc-MAA passage to be higher during exercise than at rest. Instead, we found it to be greatest with resting hypoxia and similar between both exercise conditions.

### Methodological considerations

We have previously validated the existence of IPAVs in intact rats and dogs and isolated rat, baboon, dog, and human lungs using solid latex particles of known diameter [5], [7], [29]. Although the lung is not permeable to solid latex particles 15–70 µm under resting conditions, these large particles traverse the lung with the addition of hypoxia or exercise [5], [29]. A limitation of the study of IPAVs in humans is that, in contrast to our animal studies, we are unable to directly measure the size of the ^99m^Tc-MAA particles that traverse the lung. However, we previously have demonstrated that the fraction of ^99m^Tc-MAA particles that traverse the lung in exercising humans is similar to the fraction of radiolabeled latex particles that traverse the lungs of dogs performing similar exercise (1.3 and 1.4%, respectively) [4].

It has been suggested that small particles traverse the lung by “squeezing” through distended pulmonary capillaries when pulmonary vascular pressure is elevated [15], [16]. Assuming that the pulmonary vascular distends 2% per mmHg increase in pulmonary vascular pressure [30], at physiological perfusion pressures we would expect a 6 µm pulmonary capillary to distend to no more than 8 µm. Manohar and Goetz demonstrated that the lung of the thoroughbred race horse, which apparently lacks IPAVs but is perfused at extraordinarily high pressure during exercise, is not permeable to 15µm microspheres, suggesting that the maximal capillary diameter is <15 µm [31]. Furthermore, the binding of ^99m^Tc to albumin is proportional to the particle mass and our previous calculations demonstrate that the contribution of the additional ^99m^Tc-MAA particles ≤15 µm that might pass through pulmonary capillaries during exercise is <0.4% of the total signal. [4]. Thus, we are confident that the additional ^99m^Tc signal with exercise and hypoxia is the result of large diameter particles traversing IPAVs. Furthermore, if ^99m^Tc-MAA is traversing the pulmonary capillaries, we would expect that the passage of ^99m^Tc-MAA would be greatest during hypoxic exercise when pulmonary vascular pressures are highest and to increase in all participants. Instead, we found passage to be the greatest during resting hypoxia, suggesting that distended capillaries are not the primary source of transpulmonary capillary passage.

The excellent repeatability of the measurement in resting, normoxic individuals gives us confidence that the differences observed with hypoxia and exercise represent true increases in transpulmonary particle passage and are not caused by free pertechnate or inter-injection variability. Indeed, we took care to perform all scans within 10 minutes of the conclusion of exercise and found very little free label (<0.1% of the total extrapulmonary signal), in the bladder, gut, and thyroid, where free pertechnate accumulates. If our findings were caused by free pertechnate, we would expect to find substantial labeling of these organs [32]. It is also attractive to consider filtering very small particles from the injectate. However, in our hands this resulted in the significant aggregation of particles, resulting in the formation of large particles that are unsafe for injection in humans.

### What influences the recruitment of large diameter IPAVs?

In humans and experimental animal models with lesions that result in unventilated lung regions, increasing cardiac output typically increases physiological intrapulmonary shunt (Q_S_/Q_T_) [23], [33]–[38]. It might, therefore, seem surprising and counterintuitive that we found a decrease in IPAVs perfusion with hypoxic exercise, where the cardiac output is higher, compared to rest. Our finding are, in fact, congruent with previous work in this area and understanding the interaction between FIO_2_, cardiac output, and Q_S_/Q_T_ provides insight into what factors may regulate IPAVs perfusion.

Many previous studies have relied on models of lung injury where the FIO_2_ is normoxic or hyperoxic and injured regions of the lung are focally hypoxic because of the poor ventilation (ie, respiratory distress syndrome, pulmonary edema and atelectasis, arteriovenous malformation and methacholine challenge) [33]–[38]. However, Wagner, et al. measured the increase in Q_S_/Q_T_ in a methacholine-challenged dog model with normoxic, hyperoxic, and hypoxic ventilation [39], thereby fundamentally improving our understanding of this phenomenon. In all conditions, methacholine challenge resulted in an initial 28% shunt. With normoxic and hyperoxic ventilation, this shunt increased 7% and 11% per L/min increase in cardiac output, but only 2% with hypoxic ventilation. The authors explain these different changes in Q_S_/Q_T_ by asking the reader to consider a lung with two vascular compartments with parallel resistances – one that is normal and well-ventilated and one that is injured and poorly ventilated. Under normoxic and hyperoxic conditions, the poorly ventilated compartment is hypoxic, resulting in a high degree of vasoconstriction and high vascular resistance. The resistance in the well-ventilated compartment is lower than the injured compartment. As cardiac output increases, there is a greater fall in resistance in the poorly ventilated compartment relative to the well-ventilated compartment, thereby redistributing the cardiac output to the injured compartment. When both compartments are hypoxic, the resistances are similar and the cardiac output is distributed similarly between the compartments.

To understand our finding that IPAVs perfusion decreases with exercise, we would similarly ask the reader to consider a lung with two vascular compartments. Unlike Wagner et al.’s model, these resistances are morphometrically distinct. One compartment contains large diameter of IPAVs, which lie upstream of a second compartment of pulmonary arteries, capillaries, and veins. We have previously shown in rats that IPAVs are ≥70 µm [6], placing them early in the vascular tree and upstream of the primary resistance arterioles [40] that constrict in response to hypoxia. This location is further supported by anatomic studies that identify arteriovenous connections early in the vascular tree [41]. With resting hypoxia, large diameter IPAVs are open and there is also considerable vasoconstriction of the resistance arterioles, directing blood flow through IPAVs. With exercise, the post-IPAVs resistance falls tremendously [42]. Thus, following similar logic to that used by Wagner, et al. [39], we would expect the perfusion to IPAVs to fall as a function of the fall in resistance of the non-IPAVs compartment.

The strong correlation between the transpulmonary passage of ^99m^Tc-MAA during hypoxic rest and hypoxic exercise supports this interpretation and suggests that when IPAVS are open, the fraction of the cardiac output that perfuses them is determined by two factors – 1) their relative abundance, which contributes to intersubject variability and 2) the downstream resistance, which redistributes blood flow through IPAVs and contributes to intercondition variability. Moreover, Tobin used a mechanism very similar to that we propose to identify IPAVs in isolated lungs [41]. By strategically elevating the downstream vascular resistance, he was able to preferentially fill these pathways with casting material. If IPAVs are located upstream of the vessels that are the largest contributors to the vascular resistance [43]–[45], then the tone of the resistance vessels could determine flow through IPAVs.

Eldridge, et al. [1] previously speculated that IPAVs exist as a “pressure relief valve” to normalize pulmonary pressure in the face of high cardiac output. Although this explanation seems physiologically plausible, it is not supported by these data and challenges our own previous ideas about IPAVs recruitment. It is possible that the recruitment of IPAVs is not caused by increased cardiac output and pressure, but by biochemical signals that directly impact their tone. Hyperoxia consistently prevents or reverses the recruitment of IPAVs, even in the face of high intensity exercise [12] and epinephrine and dobutamine infusion [13], suggesting that IPAVs regulation by hyperoxia occurs as a result of a distinct and secondary O_2_-mediated mechanism. Further supporting a role for oxygen sensitivity, we found previously that PvO_2_<22 mmHg is required for the recruitment of IPAVs in rats and we speculated that IPAVs are recruited via an oxygen-sensitive mechanism, in response to pulmonary arterial hypoxemia [6]. Epinephrine, dobutamine, and dopamine are vasoactive and may affect the tone of IPAVs independently of their effect on total pulmonary vascular flow. Isolated lung models may be valuable in future investigations, allowing the evaluation of biochemically-mediated effects while controlling pulmonary vascular pressure and flow.

### The A-aDO_2_ is related to the magnitude of transpulmonary ^99m^Tc-MAA passage

Whether IPAVs contribute to exercise-induced arterial hypoxemia [46] has been a controversial question [47], [48]. We have been motivated to use particles to quantify the recruitment of large diameter pathways that could contribute to anatomic shunt by the recognition of substantial pre-capillary gas exchange [49], [50] that might confound measurements made with SF_6_ or 100% O_2,_ thereby underestimating shunt under normoxic or hypoxic conditions. Although ^99m^Tc-MAA passage has been related to SpO_2_, it has not been related to the A-aDO_2_. Thus, as a secondary endpoint of this study, we measured arterial blood gases in our participants and found the magnitude of IPAVs recruitment to be well-correlated with the A-aDO_2_. Indeed, we were intrigued by the fact that the transpulmonary passage of ^99m^Tc-MAA was quite similar to actual values of physiological shunt (Q_S_/Q_T_) reported elsewhere. Vogiatzis, et al. reported Q_s_/Q_T_ in humans performing normoxic exercise at power outputs similar to ours and found Q_s_/Q_T_ to be 3.5% [51]. This is similar to the 2.5% passage of ^99m^Tc-MAA reported here. This is also similar to the amount of shunt that Gledhill et al. calculated [52] would be required to explain the remaining A-aDO_2_ after accounting for ventilation-perfusion inequality. This suggests that the shunting of venous admixture through intrapulmonary pathways may, in fact, be an important contributor to Q_s_/Q_T_ in normoxic exercise. However, we acknowledge that an important limitation of our work, and indeed the field at this point, is the inability to prevent IPAVs recruitment during exercise in an O_2_-independent manner, thereby allowing the contribution of IPAVs to the A-aDO_2_ to be evaluated during exercise without changes in FIO_2_ that might alter IPAVs patency. We also recognize that it is possible that the increase in ^99m^Tc-MAA occurs only coincidentally with the increased in A-aDO_2_.

We note a similar increase in IPAVs recruitment proportional to the increase in A-aDO_2_ in both normoxia and hypoxia, but the relative contributions of ventilation-perfusion inequality, diffusion limitation and shunt to the increased A-aDO_2_ during exercise is certainly dependent on the FIO_2_. Vogiatzis, et al. found a seven-fold increase in Q_s_/Q_T_ with hypoxic exercise, yet we found no additional increase in IPAVs recruitment with our particle method. If we speculate that IPAVs do contribute to Q_s_/Q_T,_ then additional contributors, including diffusion limitation, would need to account for the remaining A-aDO_2_. While we note a positive relationship between A-aDO2 and the fractional IPAVs recruitment, future studies will be needed to separate the simultaneous contributions of IPAVs, ventilation-perfusion inequality, and diffusion limitation in normoxia and hypoxia, posing an interesting challenge to investigators motivated to understand the mechanism of the widened A-aDO_2_ in exercising humans.

It may seem counterintuitive that the A-aDO_2_ would be unchanged with resting hypoxia, despite the fact that the IPAVS recruitment was greatest in this condition. This is likely the result of the narrow arteriovenous oxygen difference in this condition. Relying on historical data obtained from resting humans breathing FIO_2_ = 0.10 [53], we can estimate that the mixed venous saturation of hemoglobin (SO_2_) is 50% in our participants. Using our participants own mean value of alveolar PO_2_ and the Kelman equation to convert between PO_2_ and SO_2_ [54], we can estimate venous and alveolar oxygen content [27]. Using the standard shunt equation [27], we can then calculate the contribution of a 5% anatomic shunt on the A-aDO_2_. Indeed, this calculation reveals that the impact of a 5% anatomic shunt on the A-aDO_2_ is trivial under these conditions (1.2 mmHg), thereby demonstrating that IPAVs recruitment need not always result in a widened A-aDO_2_.

We appreciate that, because of the number of visits required for the completion of this study, blood gases were obtained via arterial puncture preceding the resting injections rather than by placing an in-dwelling catheter. Our participants found this to be more comfortable in the supine position, which may impact the A-aDO_2_ and limit our ability to interpret these data. There was, however, no significant difference between the A-aDO_2_ measured during the resting visit in the supine position and the A-aDO_2_ measured at rest in the upright position during the exercising visits.

### Conclusion

Here, we demonstrate the perfusion of IPAVs is greatest in hypoxic individuals and that elevating the cardiac output with exercise does not increase IPAVs recruitment.
